# Associations between autism spectrum disorder and eating disorders with and without self-induced vomiting: an empirical study

**DOI:** 10.1186/s40337-020-00359-4

**Published:** 2021-01-06

**Authors:** Noriko Numata, Akiko Nakagawa, Kazuko Yoshioka, Kayoko Isomura, Daisuke Matsuzawa, Rikukage Setsu, Michiko Nakazato, Eiji Shimizu

**Affiliations:** 1 Research Center for Child Mental Development, Chiba University, 1-8-1 Inohana, Chuo-ku, Chiba, 260-8670 Japan; 2 Department for School of Human and Social Sciences, Fukuoka Prefectural University, Fukuoka, Japan; 3 Centre for Psychiatry Research, Department of Clinical Neuroscience, Karolinska Institutet, Stockholm, Sweden; 4 Department of Cognitive Behavioral Physiology, Graduate School of Medicine, Chiba University, Chiba, Japan; 5 Koutokukai Sato Hospital, Yamagata, Japan; 6 Department of Psychiatry, Graduate School of Medicine, International University of Health and Welfare, Chiba, Japan; 7 Department of Psychiatry, Graduate School of Medicine, Chiba University, Chiba, Japan

**Keywords:** Eating disorder, Subtype, Autism spectrum disorder, Self-induced vomiting, Autism-Spectrum quotient

## Abstract

**Background:**

Although approximately 23% of anorexia nervosa (AN) patients have concomitant autism spectrum disorder (ASD), it is clinically difficult to determine ASD coexistence in patients with eating disorders. Restrictive AN is more common in younger patients and self-induced vomiting usually appears during adolescence/young adulthood, in order to prevent gaining weight caused by overeating. However, some patients are tolerant of weight gain even if they start overeating. It is important to understand the essential difference between those who vomit and those who do not vomit. In this study, we hypothesised that the absence of self-induced vomiting may be associated with the presence of ASD and aimed to assess the presence of ASD traits in each eating disorder (EDs). Clarifying this association helps to consider the coexistence of ASD in the clinical setting and can lead to the next detailed ASD evaluation, and as a result, helps to determine the appropriate treatment and support individually.

**Methods:**

We retrospectively evaluated 43 females aged 15–45 years who attended Chiba University Hospital between 2012 and 2016 using the Eating Disorder Examination Questionnaire (EDE-Q) and Autism-Spectrum Quotient (AQ) to quantify the severity of the EDs and to identify whether ASD traits were present.

**Results:**

There was no difference in the AQ score between bingeing-purging type AN and restricting type AN. However, there was significant difference in the AQ score between bulimia nervosa and binge EDs (BED). Of the 4 ED subtypes, BED had the highest ASD traits. The non-vomiting group with illness duration < 4 years had a significantly higher AQ communication score than the vomiting group with illness duration ≥4 years.

**Conclusions:**

There was a difference in the AQ score by the presence or absence of self-induced vomiting. The results of this study suggest an association between high scores on AQ and non-vomiting. Thus, evaluation of patients for the absence of self-induced vomiting while assessing them for EDs may help us to understand the association with ASD traits.

## Plain English summary

Although about 23% of anorexia nervosa (AN) patients have concomitant autism spectrum disorder (ASD), it is clinically difficult to determine ASD coexistence in patients with eating disorders (EDs). Restrictive AN is more common in younger patients and self-induced vomiting typically appears during adolescence, in order to prevent gaining weight caused by overeating. However, some patients state that they ‘would never ‘want to vomit’ and are tolerant of weight gain even if they start overeating.

We aimed to assess the presence of ASD traits in each subtype of ED and explore whether an association exists between ASD traits and EDs with or without self-induced vomiting. We retrospectively evaluated 43 females aged 15–45 years using the Eating Disorder Examination Questionnaire (EDE-Q) and Autism-Spectrum Quotient (AQ) to quantify the severity of the ED and to identify whether an ASD trait was present.

The AQ tended to be higher in the group without than in the group with self-induced vomiting. Patients with binge EDs (BED) had the highest AQ score. The results of this study suggest an association between high scores on AQ and non-vomiting. In case of absence of vomiting in EDs, the presence of ASD and a change in the treatment course must be considered. Further verification is required in the future.

## Background

According to the Diagnostic and Statistical Manual of Mental Disorders, Fifth Edition (DSM-5), eating disorders (EDs) can be classified into anorexia nervosa (AN), bulimia nervosa (BN), and binge eating disorder (BED) [1, 2]. AN can then be further divided into a restricting (AN-R) type and a binge eating with self-induced vomiting (AN-BP) type. Patients frequently transition from one ED type to another, typically from the AN-R type to the AN-BP type [3]; this may be attributed to the fact that strict dietary restrictions cannot be maintained for a long time.

It is interesting that many of the common features of EDs are similar to the cognitive rigidity in the presence of changing environmental demands that is often seen in autism spectrum disorder (ASD) [4]. This may indicate a pathological link between the two disorders, with some studies indicating that 18–23% of patients with AN have comorbid ASD [5–7]. Other research has shown that scores on the Eating Disorder Examination Questionnaire (EDE-Q) were significantly and positively correlated with those on the Autism-Spectrum Quotient (AQ), 10-item version, but not with the body mass index (BMI) [8]. This correlation has also been observed in studies conducted on adults and adolescents with AN [9, 10]. Studies have shown that prognosis may be worse when EDs and ASD are comorbid [4, 5, 11].

It is necessary to assess ASD traits when deciding the treatment policy for patients with EDs. However, clinically, it is difficult to determine the ASD traits of patients with EDs. Restrictive AN is more common in younger patients, and self-induced vomiting usually appears during adolescence/young adulthood in order to prevent gaining weight caused by overeating. However, we clinically observed some patients who say that they ‘would never want to vomit’ and are tolerant of weight gain even if overeating began. We aimed to determine the difference between those who vomit and those who do not vomit and thought that this difference might be related to the ASD trait. Therefore, we decided to investigate whether the presence or absence of vomiting was associated with the ASD trait.

To date, many studies have discussed links between ASD and AN restricting type [8]. Given that many patients transit between ED subtypes, it is logical that research should be more expanded to other subtypes of ED. In this study, we hypothesised that the absence of self-induced vomiting may be associated with the presence of ASD and aimed to assess the presence of ASD traits in each ED. Clarifying this association helps to consider the coexistence of ASD in the clinical setting and can lead to the next detailed ASD evaluation, and as a result, helps to determine the appropriate treatment and support individually.

## Methods

### Procedure

The data were retrospectively sampled from outpatients from clinical records when they participated in one of three independent studies on ED were conducted at Chiba University Hospital between 2012 and 2016. The Institutional Ethics Committee of Chiba University Graduate School of Medicine approved this study (no. 3431). All subjects provided written informed consent.

Subjects were diagnosed by a psychiatrist with experience in EDs, using the DSM-IV revised criteria [12] and the DSM-5 [2]. AN, BN and BED were included if the criteria was fully met; however, one atypical ED who was chewing type was excluded from this study. Self-report questionnaires were completed by subjects at that time visited the hospital.

### Subjects

The collected sample size was 43, all were female. Among them, 42 subjects were finally analysed because the chewing type was excluded. Among those 42 subjects, 23 of BN, 8 of AN-BP, 6 of AN-R and 5 of BED were included. The subjects with self-induced vomiting (BN and AN-BP) were 31, and those without self-induced vomiting (AN-R and BED) were 11 at the time of assessment. The mean age of the 42 subjects was 26.2 (± 7.8). Vomiting was assessed by both a psychiatrist and self-reported questionnaire (EDE-Q).

### Measures

Relevant demographic data were collected, including age, duration of illness and BMI. ED severity was assessed using the global EDE-Q score [13], whereas autistic tendencies were assessed using the AQ [14].

#### Eating disorder examination questionnaire (EDE-Q)

The EDE-Q is a standardised and well-validated 36-item self-report questionnaire that measures the severity of ED symptoms and behaviours in the 28 days leading up to the survey [13]. In the questionnaire, patients are asked to rate how often they have engaged in specific ED behaviours or held ED concerns during the previous 28 days. The questionnaire generates scores for four subscales—‘dietary restraint,’ ‘weight concern,’ ‘shape concern,’ and ‘eating concern’—together with a global score that reflects overall illness severity. The maximum global score is 6, with higher scores indicating greater severity. The optimal cut-off score is 2.5 for discriminating between those with the disorder and healthy controls [15]. Cronbach’s α ranged from.78 (Eating concern at Time 1) to.92 (Shape Concern at Time 2) for women [16]. In the Japanese version, Cronbach’s α coefficient was 0.94 for the global scale, 0.81 for the restricting subscale, 0.86 for the eating concern subscale, 0.88 for the shape subscale, and 0.79 for the weight subscale. It showed a significant correlation with the Eating Attitude Test-26 and the Eating Disorder Inventory-91 [17]. Considering the findings of previous studies [13, 18], the EDE-Q appears to be a psychometrically sound self-report measure for the screening of EDs.

#### Autism-Spectrum quotient (AQ)

The 50-item AQ was developed to provide a brief self-report measure of autistic traits in adults but was not designed to be used as a diagnostic tool despite its widespread use. The AQ consists of five domains associated with ASDs: social skills, attention switching, attention to detail and communication and imagination. Each question allows the subject to indicate ‘definitely agree,’ ‘slightly agree,’ ‘slightly disagree,’ or ‘definitely disagree.’ Approximately half the questions are worded to elicit an ‘agree’ response and half to elicit a ‘disagree’ response in neurotypical individuals. The cut-off score for ASD is 33. The internal consistency of items in each of the five domains was also calculated, and Cronbach’s α coefficients were moderate-to-high (Communication = .65; Social = .77; Imagination = .65; Local Details = .63; and Attention Switching = .67) [14, 19]. In the Japanese version, the global scale was α = 0.81. The α coefficient for each individual scale was as follows: 0.78 for social skills, 0.63 for attention switching, 057 for attention to detail, 0.64 for communication, and 0.51 for imagination [19].

### Statistical analysis

All data were reported as means and standard deviations or numbers (number of people) and percentages as appropriate. Demographic data were analysed by Kruskal–Wallis analysis and multiple comparisons were performed by the Steel–Dwass method. In addition, AQ scores and EDE-Q scores of patients with and without self-induced vomiting and EDE-Q scores were compared using the Mann–Whitney test, because we had assumed that the variables would not be normally distributed owing to the relatively small sample size. Assuming that BMI is a confounding factor, Mann–Whitney test was performed between anorexic group and bulimic group. Furthermore, illness duration can be a confounding factor; therefore, Kruskal–Wallis analysis was conducted to confirm this. (Correlation analysis could not be performed owing to the relatively small sample size). Finally, the ratio of the subtypes and the number of patients who exceeded the cut-off value of the AQ score was determined, and the difference in the ratio of the number of patients was examined by Fisher’s exact test. We also calculated the effect size using Cramer’s V. A Cramer’s V > 0.10 was used as the criterion for a small effect, a value > 0.30 as a medium effect, and > 0.50 as a large effect (http://jspt.japanpt.or.jp/ebpt_glossary/effect-size.html). There was no missing value. There were some outlier values; however, all the numbers were clinically possible and were not excluded. Statistical analyses were performed using the STAT statistical package and js-STAR version 9.7.8j.

## Results

### Demographic analysis and clinical characteristics in subtypes of ED

The 42 female outpatients aged 12–45 years (mean 26.2 ± 7.8 years) were analysed. The sample comprised the following diagnoses: 23 with BN (54.7%), 8 with AN-BP (19.0%), 6 with AN-R (14.3%), 5 with BED (11.9%). Among these patients, only 11 did not have self-induce vomiting (i.e. had AN-R and BED) at the time of visiting the hospital. The clinical and demographic characteristics are summarised in Table 1.

**Table 1:** Clinical profiles of the 42 patients

	BN	AN-BP	AN-R	BED	Kruskal-Wallis analyses	
	*n* = 23	*n* = 8	*n* = 6	*n* = 5	H	Multiple Comparisons by Steel-Dwass
	Mean (SD)	Mean (SD)	Mean (SD)	Mean (SD)		
Illness duration (years)	6.4 (5.8)	10.0 (6.7)	1.9 (1.7)	4.6 (3.7)	8.2*	AN-BP > AN-R
BMI	20.4 (2.4)	17.0 (0.7)	15.6 (1.5)	24.3 (7.0)	26.3**	BN > AN-BP,BN > AN-R, AN-BP < BED, AN-R < BED
Age (years)	27.0 (7.0)	28.1 (7.0)	18.6 (4.8)	28.7 (11.2)	8.3*	N.S
EDE-Q						
Diagnosis	74.5 (40.8)	78.8 (39.4)	15.2 (11.5)	62.2 (45.8)	12.8**	BN > AN-R, AN-BP > AN-R
Restricting	3.4 (1.7)	5.2 (1.0)	2.4 (1.5)	4.0 (1.6)	10.7*	AN-BP > AN-R
Eating concern	3.4 (1.5)	4.9 (0.6)	2.0 (1.4)	3.8 (1.9)	10.9*	AN-BP > AN-R
Weight	4.4 (1.4)	5.4 (0.6)	2.6 (1.9)	5.2 (0.5)	10.2*	AN-BP > AN-R
Shape	4.6 (1.4)	5.1 (0.7)	2.8 (1.8)	5.3 (0.5)	6.6	N.S
Global Score	3.9 (1.3)	5.0 (0.5)	2.4 (1.6)	4.5 (0.9)	10.5*	AN-BP > AN-R
AQ						
Total score	22.1 (6.4)	25.3 (4.8)	26.0 (7.5)	32.4 (6.2)	7.6	N.S
Social skills	4.7 (2.5)	5.1 (2.6)	5.7 (3.6)	8.0 (1.0)	7.8*	N.S
Attention Switching	5.7 (1.9)	7.1 (1.6)	6.2 (1.8)	6.4 (1.5)	2.9	N.S
Attention to detail	3.5 (2.0)	5.3 (3.3)	4.0 (1.7)	7.6 (2.5)	9.4*	BN < BED
Communication	3.8 (2.3)	4.4 (1.1)	6.0 (2.1)	6.2 (1.3)	8.7*	N.S
Imagination	4.4 (2.0)	3.4 (1.8)	4.2 (1.5)	4.2 (2.2)	2.2	N.S

*BN* Bulimia Nervosa, *AN-BP* Anorexia Nervosa Binge Purging, *AN-R* Anorexia Nervosa Restricting, *BED* Binge Eating Disorder, *BMI* Body Mass Index, *EDE-Q* Eating Disorder Examination Questionnaire, *AQ* Autism Spectrum Quotient

**p < 0.01, *p < 0.05

As shown in Table 1, the groups were not significantly different in their age, there was a significant difference in illness duration between AN-BP and AN-R (AN-BP: 10.0 ± 6.7, AN-R: 1.9 ± 1.7). However, the AN groups (AN-BP and AN-R) had a significantly lower BMI compared with the other groups (BN and BED) (AN-R: 15.6 ± 1.5, AN-BP: 17.0 ± 0.7; BN: 20.4 ± 2.4, BED: 24.3 ± 7.0) (H = 26.3, *p* < 0.01). There were also statistically significant differences between the AN groups and BN in terms of the EDE-Q global scores for clinical severity (AN-R: 15.2 ± 11.5; BN: 74.5 ± 40.8; and AN-BP: 78.8 ± 39.4). AN-BP tended to be higher than the other subtypes in terms of restricting, eating and weight, that were the sub-items of EDE-Q, and there was a significant difference in the comparison with AN-R (Restricting: AN-BP 5.2 ± 1.0, AN-R 2.4 ± 1.5; Eating: AN-BP 4.9 ± 0.6, AN-R 2.0 ± 1.4, Weight: AN-BP 5.4 ± 0.6, AN-R 2.6 ± 1.9).

Table 1 also shows that the average of AQ total score was highest for patients with BED (32.4 ± 6.2), followed by those with AN-R (26.0 ± 7.5), AN-BP (25.3 ± 4.8), and BN (22.1 ± 6.4). The difference between BN and BED was significant for the attention to detail score (BN: 3.2 ± 2.0, BED: 7.6 ± 2.5) (H = 9.4, *p* < 0.05).

### Clinical characteristics, EDE-Q and AQ score by the presence or absence of self-induced vomiting

As observed in Table 2, two groups were formed: 31 patients with self-induced vomiting and 11without self-induced vomiting. Although there were no significant differences in age or BMI, there was a significant difference in illness duration between vomiting present group and vomiting absent group (BN, AN-BP: 7.3 ± 6.2, AN-R, BED: 3.1 ± 3.0).

**Table 2:** Comparison of AQ scores by the presence or absence of self-induced vomiting (*n* = 42)

	BN,AN-BP Vomiting(+)	AN-R,BED Vomiting(−)	Mann–Whitney	
	*n* = 31	*n* = 11		
	Mean (SD)	Mean (SD)	*U*	*r*
Illness duration (years)	7.3 (6.2)	3.1 (3.0)	100.5*	0.31
BMI	19.5 (2.6)	19.6 (6.5)	132.5	
Age (years)	27.3 (6.9)	23.2 (9.5)	111.0	
EDE-Q				
Diagnosis	75.6 (39.8)	36.5 (38.8)	67.5**	0.46
Restricting	3.9 (1.7)	3.1 (1.7)	116.0	
Eating concern	3.8 (1.5)	2.8 (1.8)	112.0	
Weight	4.6 (1.3)	3.7 (1.9)	123.5	
Shape	4.7 (1.3)	3.9 (1.8)	140.0	
Global Score	4.2 (1.2)	3.4 (1.7)	120.0	
AQ				
Total score	22.9 (6.1)	28.9 (7.4)	102.5	
Social skills	4.8 (2.5)	6.7 (2.9)	89.5*	0.36
Attention Switching	6.0 (2.0)	6.3 (1.6)	153.0	
Attention to detail	3.9 (2.5)	5.6 (2.7)	103.0	
Communication	4.0 (2.1)	6.1 (1.7)	71.5**	0.44
Imagination	4.2 (2.0)	4.2 (1.7)	166.0	

*AN* anorexia nervosa (either -R for restricting or -BP for binge eating with self-induced vomiting), *AQ* Autism Spectrum Quotient, *BMI* body mass index, *BN* bulimia nervosa, *BED* binge eating disorder, *EDE-Q* Eating Disorder Examination Questionnaire.

**p < 0.01 *p < 0.05

There were no significant differences in the EDE-Q, except for the ‘diagnosis’ category, which is expected to be affected by the frequency of self-induced vomiting. The AQ total scores of those who did not self-induced vomiting were significantly higher than for those who self-induced vomiting. In particular, the scores for social and communication skills—which are subscales of the AQ—were significantly higher in the group that did not have self-induced vomiting.

There were also statistically significant differences between the vomiting and non-vomiting groups in the EDE-Q global scores for clinical severity. Two bulimic vomiting subtypes (BN and AN-BP) scored higher than non-vomiting subtypes (U = 67.5, *p* < 0.01, r = 0.46).

However, BMI was also considered to be a confounder of EDE-Q and AQ scores; therefore, the patients were divided by anorexic and bulimic, so only AN-BP to AN-R and BN to BED were analysed (Table 3).

**Table 3:** Comparison AN-BP to AN-R and BN to BED (n = 42)

	Anorexia nervosa (*n* = 14)	Bulimia nervosa (*n* = 28)
	(AN-BP vs AN-R)	(BN vs BED)
	p (Mann-Whitney)	p (Mann-Whitney)
Illness duration (years)	0.00*	0.83
BMI	0.05*	0.17
Age (years)	0.01*	0.61
EDE-Q		
Diagnosis	0.00*	0.47
Restricting	0.01*	0.65
Eating concern	0.00*	0.7
Weight	0.01*	0.31
Shape	0.03*	0.25
Global score	0.00*	0.42
AQ		
Total score	0.8	0.01*
Social skills	0.6	0.00*
Attention switching	0.47	0.39
Atention to detail	0.6	0.00*
Communication	0.11	0.03*
Imagination	0.32	0.88

*AN* anorexia nervosa (either -R for restricting or -BP for binge eating with self-induced vomiting), *AQ* Autism Spectrum Quotient, *BMI* body mass index, *BN* bulimia nervosa, *BED* binge eating disorder, *EDE-Q* Eating Disorder Examination Questionnaire.

*p < 0.05

Despite the significant differences in illness duration, BMI and age between AN-BP and AN-R, there were no differences in any of the AQ sub-items. In contrast, there was no significant difference in the illness duration, BMI and age between BN and BED. However, there was a significant difference in the social skills, attention to detail and communication in the AQ score.

In addition, illness duration could be a confounding factor for the EDE-Q and AQ scores. Therefore, we performed the multivariate analysis with 4 groups: illness duration of < 4 years for patients with and without vomiting and illness duration of ≥4 years for patients with and without vomiting. A Kruskal–Wallis analysis was conducted to confirm these groups, because illness duration of ≤4 years or more has been the common cut-off used for determining acute vs chronic EDs [20, 21].

There were 22 patients (illness duration ≥4 years) and 9 patients (illness duration < 4 years) in the vomiting group and 3 patients (illness duration ≥4 years) and 8 patients (illness duration < 4 years or less) in the non-vomiting group. The results are shown in Tables 4 and 5. Illness duration resulted in significant differences in AQ communication, EDE-Q diagnosis and age. The communication score of AQ was highest in the non-vomiting group with illness duration < 4 years, followed by the non-vomiting group with illness duration ≥4 years. The communication score of AQ in the non-vomiting group with illness duration < 4 years was significantly higher than that in the vomiting with illness duration ≥4 years.

**Table 4:** Multivariate analysis of AQ and EDE-Q of 4 groups in illness duration with Kruskal-Wallis analysis

AQ:Communication			statistic H = 8.38			df = 3, *p* < .05
Group	N	Mean	SD	Max	Min	Average of the rank
1	9	3.78	2.1	7	0	17.83
2	8	6.25	1.48	9	4	31.44
3	22	4.05	2.03	9	0	18.5
4	3	5.67	1.89	7	3	28

== Multiple Comparisons by Steel-Dwass == (* *p* < .05)

A2 > A3 * (T = 2.656)

Group1: < 4 years of illness duration, Vomiting(+); Group2: < 4 years of illness duration, Vomiting(−)

Group3: ≥4 years of illness duration, Vomiting(+); Group4: ≥4 years of illness duration, Vomiting(−)

*N* Number of subjects, *Mean* Mean value, *SD* Standard Daviation, *Max* Maximum value, *Min* Minimun value

*df* degrees of freedom

**Table 5:** Multivariate analysis of AQ and EDE-Q of 4 groups in illness duration with Kruskal-Wallis analysis

EDE-Q: Diagnosis			statistic H = 12.44			df = 3, *p* < .01
Group	N	Mean	SD	Max	Min	Average of the rank
1	9	75.44	37.27	141	21	24.78
2	8	21.38	13.9	46	4	7.75
3	22	75.68	39.9	139	10	24.84
4	3	77	47.55	132	16	23.83

== Multiple Comparisons by Steel-Dwass == (* p < .05)

A1 > A2 * (T = 3.081)

A2 < A3 * (T = 3.332)

Group1: < 4 years of illness duration, Vomiting(+); Group2: < 4 years of illness duration, Vomiting(−)

Group3: ≥4 years of illness duration, Vomiting(+); Group4: ≥4 years of illness duration, Vomiting(−)

*N* Number of subjects, *Mean* Mean value, *SD* Standard Daviation, *Max* Maximum value, *Min* Minimun value

*df* degrees of freedom

### AQ score cut-off value by ED subtype

Lastly, the ratio of the subtypes and the number of patients who exceeded the cut-off value of the AQ score were examined, and the difference in the ratio of the number of patients was examined using Fisher’s exact test. Three of eight patients of BED had an AQ score above 33, which well exceeded expected value in 60% of patients with BED. By contrast, one patient with BN had an AQ ≥33 (4.3%), and this amount was below the expected value. The difference between the BN and BED groups was significant (*p* = 0.02) (Table 6).

**Table 6:** Number and ratio of people who exceeded the AQ cut-off value by ED subtype (n = 42)

	BN	AN-BP	AN-R	BED	Fisher	Cramer’s
AQ score	n (%)	n (%)	n (%)	n (%)	*χ2*	V
AQ≧33	1 (4.3)	1 (12.5)	1 (16.7)	3 (60.0)	10.4*	0.50
	3.29	1.14	0.86	0.71		
AQ≦32	22 (95.7)	7 (87.5)	5 (85.7)	2 (40.0)		
	19.71	6.86	5.14	4.29		

*AN* anorexia nervosa (either -R for restricting or -BP for binge eating with self-induced vomiting), *AQ* Autism Spectrum Quotient, *BN* bulimia nervosa, *BED* binge eating disorder.

*p < 0.05

## Discussion

The aim of the current study was to examine the relationship between each subtype of EDs and ASD trait regarding the presence or absence of self-induced vomiting. We first compared the illness duration, BMI, EDE-Q scores, and AQ scores in the four groups (BN, AN-BP, AN-R, and BED). Next, the four subtypes were divided as per the presence or absence of self-induced vomiting; the illness duration, BMI, age, EDE-Q, scores and AQ scores were compared. Assuming that BMI is a confounding factor, we compared EDE-Q and AQ scores between anorexic group (AN-BP and AN-R) and bulimic group (BN and BED). Furthermore, illness duration could be a confounding factor; therefore, we performed additional analysis to confirm it. Finally, we examined the ratio of the subtypes and the number of patients who exceeded the cut-off value of the AQ score.

We had hypothesised that patients without self-induced vomiting had a higher tendency for ASD; however, there was no difference in the AQ between AN-BP and AN-R. The significant difference in the AQ score between BN and BED was clear. Of the four ED subtypes, BED had the highest ASD trait. Even after the statistical analyses were adjusted to determine confounding factors, such as low BMI and duration of illness, the significant AQ differences in our results can be explained by the difference between BN and BED. However, this finding is not completely aligned with our hypothesis that ASD traits are correlated with a lack of vomiting. That is, for EDs of patients within or above normal weight, ASD traits were associated with a lack of vomiting in this study. In addition, when the illness duration was divided into < 4 years and ≥ 4 years, non-vomiting was common in the < 4 year group and vomiting was common in the ≥4 year group. This result supports the rationale for a 4 year cut-off to designate the chronic and acute phases. Notably, early intervention in ED (before the transition from onset to vomiting) may be necessary.

In addition, the AQ communication score was significantly higher in the group without vomiting and with < 4 years of illness duration than in the group with vomiting ≥4 years of illness duration. Second, the non-vomiting group ≥4 years of illness duration scored higher than the vomiting groups. This result suggested that the non-vomiting groups tended to have a higher AQ communication score. A previous study had reported that all-or-none thinking about food and dieting was typical of patients with BED [22]. Some patients say that they ‘would never want to vomit’ and are tolerant of weight gain even if they start overeating. In these BED patients, the absence of self-induced vomiting means that they tend to be obese [19], and it is unclear why they do not vomit in the face of weight gain [21]. If considering the characteristics of ASD, it may be possible that some patients with BED do not vomit because another obsessive compulsion arising from ASD is stronger than the core psychopathology of the ED, i.e. fear of being fat and the failure of severe restriction leads to acceptance of weight gain. This is seen clinically in our practise with comments from patients such as ‘I am scared to vomit,’ ‘My life is over when I am vomiting,’ or ‘Looking at vomit disgusts me.’ In such instances, the fear or aversion to vomiting might be stronger than the desire not to gain weight. In addition, patients who were absorbed in dietary restrictions were able to postpone the desire to lose weight due to the disgust of vomiting along with the failure of restrictions, and to endure the weight gain somehow could not be explained by the psychopathology of EDs. In some cases, from the experience of vomiting once in the past, people have a strong sense to visceral sensations and/or disgust of vomiting; they are unable to forget the trauma and find it difficult to eat food because they do not want to vomit again. People with hyperaesthesia within the autism spectrum are reluctant to induce vomiting. In addition, some patients cannot eat because they are afraid of vomiting.

If there is an ASD trait, it is easy to fall into maladaptation to environment because of impairment of social skills, communication and lack of flexibility. In this study, social skills and communication scores of AQ in patients without self-induced vomiting were higher than those in patients with self-induced vomiting.

The maladaptation to environment may lead to routine behaviour, because certain routine behaviour, such as routine dietary patterns of patients, tends to reassure individuals. Environmental adjustment is the first requirement for such individuals.

For patients with EDs, it may be necessary to prioritise the identification of characteristics over the diagnosis or types. When assessing patients with EDs who never vomit, it is important to clarify the reason; i.e. determine what they are afraid of as a consequence of vomiting. Therefore, detailed assessments for appropriate recognition of each patient who does not vomit and the application of empirically derived treatments are required. Based on the above, for patients with BED with a high tendency towards ASD, environmental adjustment and psychoeducation regarding ASD may be necessary. In addition, patients should not be corrected in terms of their diet choices that are derived from sensory sensitivity (i.e. sense of smell, taste), and their feeling of disgust should not be ignored. It is also essential to take into consideration in their abnormal eating behaviour caused by the stress of their poor communication skills. For patients (AN-R and BED) who have not vomited and have illness duration of < 4 years, it is important for therapists to identify these patients’ attitude towards self -induced vomiting for the evaluation of their ASD traits and to perform an early intervention before the condition becomes chronic. To the best of our knowledge, this is the first study to compare the predisposition for ASD by ED subtype and the presence or absence of self-induced vomiting.

This study had some limitations. Of note, the sample size was small, there were differences in the number of participants in each subtype. There were no data for healthy subjects to compare patient data with standard values. Besides the above, there was a large age spread. In general, it is known that AN-R is much more common in the younger ages, and self-induced vomiting usually appear later on in adolescence/young adulthood. The differences of subjects’ age, BMI and illness duration may always be a confounder and should be controlled it with bigger sample size.

Symptoms of depression and anxiety disorders, irritability, emotional lability and obsessional features are frequent accompaniments in ED. Typically, these features worsen with weight loss and improve with weight regain [3]. Interest in the outside world also declines as patients become underweight, with the result that most patients become socially withdrawn and isolated. Since we did not measure anxiety or depression in this study, it is unclear how these were associated with ED and AQ scores. Since ED has a high incidence of anxiety and depression, it should be added to the evaluation index in the future study. There are still problems to be examined in the future regarding coexistence of autism trait and measurement of individual differences.

This study used only the AQ for evaluation of ASD traits. We should have used the AQ-10 in addition to the AQ because a few adolescent patients were included in our study. These are good tools for assessing the presence or absence of ASD traits in a busy clinical setting. However, the AQ is a self-completed scale and is not used to diagnose ASD. Using the Autism Diagnostic Observation Schedule (ADOS) or the Autism Diagnostic Interview (ADI) for ASD evaluation, excluding patients with extremely low body weights, and including control subjects in a larger overall sample should be done in future research [9, 21]. A future issue is to determine the clinical usefulness of using the ASD evaluation tool, which is called the gold standard such as ADOS and ADI, for ED patients whose ASD characteristics are considered to be a factor for maintaining the symptoms.

## Conclusions

Our results suggest that when body weight is above normal, patients with EDs without self-induced vomiting might have a higher ASD trait than those with it. In addition, when patients with EDs are divided by illness duration, among those with shorter illness duration (< 4 years), those without self-induced vomiting might have a higher ASD trait than those with it, and the difference of ASD trait between patients who do and do not vomit could be smaller among those with longer illness duration (≥4 years).

Given these results and considering that the prevalence of higher ASD tendencies in adults with EDs might contribute to the significant treatment resistance to conventional therapies [23], it is important for therapists to determine patients’ ASD trait from an early stage and to be flexible in designing treatments that are individually tailored for each patient. This study, which investigated the association between ASD traits and self-induced vomiting, could provide helpful points for future clinical research.

## Abbreviations

DSM-5: the Diagnostic and Statistical Manual of Mental Disorders, Fifth Edition
EDs: Eating disorders
AN: Anorexia nervosa
BN: Bulimia nervosa
BED: Binge eating disorder
AN-R: restricting type of AN
AN-BP: binge eating with self-induced vomiting type
ASD: Autism spectrum disorder
BMI: Body Mass Index
EDE-Q: Eating Disorder Examination Questionnaire
AQ: Autism-Spectrum Quotient
